# The Clinical Manifestations and Disease Burden of Cystinosis in Saudi Arabia: A Single-Tertiary Center Experience

**DOI:** 10.7759/cureus.52662

**Published:** 2024-01-21

**Authors:** Reem Algasem, Nedaa Zainy, Essam Alsabban, Hamad Almojalli, Syed Raza, Tariq Ali, Deiter Broering, Nawal Rubaya, Hassan Aleid

**Affiliations:** 1 Department of Pharmacy, King Faisal Specialist Hospital and Research Centre, Riyadh, SAU; 2 Department of Pediatric Nephrology, King Faisal Specialist Hospital and Research Centre, Riyadh, SAU; 3 Department of Pediatric Transplant Nephrology, King Faisal Specialist Hospital and Research Centre, Riyadh, SAU; 4 Department of Kidney and Pancreas Transplant, King Faisal Specialist Hospital and Research Centre, Riyadh, SAU

**Keywords:** saudi arabia, cystinosis, immediate release cysteamine, manifestation, complications

## Abstract

Background: There is a lack of regional and local evidence that describes the nature of cystinosis, a multiorgan accumulation of cystine, and its extent of organ damage. Therefore, this study aimed to determine the outcomes of cystinosis in patients who were followed up at a large tertiary care hospital.

Methods: Medical records of patients with cystinosis were retrospectively reviewed. Patients' baseline demographics, lab values, medications, comorbidities, and complications were collected and described. Univariable and multivariable logistics regression models were constructed to control for confounders and build prediction models.

Results: In our cohort of 39 patients, the mean age was 13.8±9.9 years. Approximately 56.4% of the patients had stunted growth, and the mortality rate was 25.6%. Regarding complications, the majority of patients developed myopathy (79.5%), end-stage renal disease (ESRD) (74.4%), and hypothyroidism (71.8%). Age (odds ratio=1.14, 95% confidence interval (95% CI): 1.012, 1.285) and stunted growth (odds ratio=6.62, 95% CI: 1.024, 42.835) were found to be predictors of renal replacement therapy and renal transplantation, respectively (p<0.047).

Conclusion: This study on cystinosis patients reveals a high incidence of renal complications, with a significant mortality rate and common complications such as myopathy and ESRD. Age was found to be an independent risk factor for renal replacement therapy, while stunted growth predicted the need for transplantation. These findings underscore the urgency for early diagnosis, comprehensive treatment, and careful monitoring in managing cystinosis effectively.

## Introduction

Cystinosis, a rare autosomal recessive disorder, is characterized by an abnormal buildup of amino acids, cystine, in cellular lysosomes [1]. Excessive cystine gets deposited in different organs, resulting in multiorgan damage, notably the kidneys [1,2]. The estimated worldwide prevalence of cystinosis is 0.001% in newborns [3]. In Saudi Arabia, 136 per million patients have been estimated to have end-stage renal disease (ESRD), with several cases being caused by cystinosis [4]. In a study at King Faisal Specialist Hospital and Research Center (KFSH&RC), 7/35 patients were found to have hereditary Fanconi syndrome due to cystinosis [5]. In contrast, Ramprasad et al. stated that cystinosis was a common cause of tubulopathies in Riyadh [6]. The exact prevalence of cystinosis in Saudi Arabia has not yet been documented; however, several small-size case series studies have discussed the morbidity of cystinosis in Saudi patients [7-9].

The diagnosis of cystinosis includes measuring white blood cell (WBC) leukocyte cystine content, whereby a value exceeding 2 nmol of half cystine per milligram of protein indicates a positive diagnosis [1,10]. Furthermore, genetic testing can be performed to determine the presence of a cystinosin, lysosomal cystine transporter (CTNS) gene mutation [11]. Signs and symptoms of cystinosis differ by age. The majority of infants (95%) develop infantile nephropathic cystinosis characterized by polyuria, polydipsia, and failure to thrive [11,12]. However, cystinosis is generally asymptomatic in neonates but can progress to cause Fanconi syndrome at 6-12 months of age, characterized by fluid and electrolyte imbalances, polyuria, polydipsia, vomiting, constipation, dehydration, rickets, and growth failure [12]. On the other hand, a small group of patients in late childhood or adolescence are diagnosed with juvenile-late-onset cystinosis [11,12]. This group of patients is mainly asymptomatic but can also present with mild renal Fanconi syndrome [12]. At older ages, cystinosis patients can experience a non-nephropathic ocular form of cystinosis, whereby cystine accumulates in the cornea and causes photophobia [13,14].

A therapeutic cure for cystinosis has not been established; however, existing therapies utilizing cysteamine levels aim to delay the progression to ESRD and extra-renal manifestation [15-17]. These therapies include immediate-release cysteamine and extended-release cysteamine. Cysteamine reduces intralysosomal cystine concentrations in various body cells and tissues [16]. Furthermore, immediate-release cysteamine eye drops have been developed to treat corneal cystinosis [14].

Cystinosis has been well-described and documented in the literature; however, there is a lack of data describing the condition's prevalence, characteristics, and prognosis in Arab countries, including Saudi Arabia. In this study, we aimed to address this shortcoming and describe the burden and complications of cystinosis among patients at the KFSH&RC.

## Materials and methods

Study design and setting

This retrospective observational study was conducted over a period of 14 months at the Department of Nephrology at KFSH&RC, Riyadh, Saudi Arabia. Data were retrospectively collected from our institution's electronic health records. This study received ethical approval from the local ethical committee of KFSH&RC (IRB No. 2181115) with a waiver of informed consent.

Inclusion and exclusion criteria

We included patients with a diagnosis of cystinosis, receiving or not receiving immediate-release cysteamine therapy (oral or eye drops), who visited the Nephrology and Transplant Clinic at KFSH&RC. Patients with incomplete data regarding their demographics, laboratory parameters, and treatment outcome were excluded. 

Outcome measurements

Our prime objective was to document the morbidity and mortality rates associated with this condition among our patient population. Initially, we compared baseline demographics between patients with chronic kidney disease (CKD), on dialysis, and renal transplant. Furthermore, a subgroup analysis was performed to compare height and weight for age percentile among patients who underwent hemodialysis or organ transplantation versus those who did not. Deceased and living patients were compared in terms of baseline demographics and laboratory values. In addition, complications in cystinosis patients were reported. Moreover, we aimed to determine pre-operative characteristics independent predictors of mortality, renal dialysis, and organ transplantation in cystinosis patients.

Data quality and collection

Data were collected by the trained staff. The data collection form was coded and entered into Microsoft Access. Patient clinical and demographic characteristics included age, gender, a positive family history of cystinosis, weight, height, presence of stunted growth, living status, city address of patients, and type of immediate-release cysteamine therapy (none, oral, drops, or both), and laboratory data were collected from the hospital's electronic system.

Statistical analysis

Categorical variables were compared using the chi-square test and presented as counts and percentages. Continuous variables were compared using the Mann-Whitney U or Kruskal-Wallis tests and presented as means and standard deviations. Univariate and multivariate logistic regression models were constructed to control for possible confounders and to build the prediction model. The significance level was set at <0.05 for all analyses. Statistical analyses were performed using Statistical Package for Social Science (SPSS, version 27; IBM SPSS Statistics for Windows, Armonk, NY).

## Results

Our cohort consisted of 41 patients with a possible diagnosis of cystinosis. After careful chart review, patients were excluded as the attending nephrologist provided no precise diagnosis of cystinosis. Hence, the study included 39 patients with a confirmed diagnosis of cystinosis. Cystinosis patients had a mean age of 13.8±9.9 years, mean weight of 39.2±15.2 kg, and mean height of 137±17.8 cm. Approximately 53.8% of patients were females.

Regarding treatment therapies, most patients were on both oral and eye drops, immediate-release cysteamine therapy (56.4%) as compared to oral therapy alone (30.8%) and eye drops alone (2.6%). Four patients (10.3%) did not use immediate-release cysteamine therapy. We could not verify the age at diagnosis and therapy initiation. Patient medical history included patients with CKD (12.8%), those on dialysis (30.8%), and patients who underwent kidney transplantation (56.4%). Those patients using ophthalmic therapy were seen by an ophthalmologist, and two (5.1%) of them underwent corneal transplantation.

A subgroup analysis was performed to compare baseline demographics between patients with compromised renal health indicated by CKD, dialysis, or renal transplant patients. Patients who underwent kidney transplantation were found to be significantly older (17.4±9.4 years) as compared to those who had CKD or were on dialysis (p=0.041). Furthermore, mean weight was significantly higher in patients with CKD (46.1 kg) compared to those on dialysis or renal transplant (p=0.046). All of those patients who were not receiving immediate-release cysteamine therapy were found to be on dialysis (25%). Baseline characteristics are shown in Table 1.

**Table 1:** Baseline characteristics for the total cohort and a comparison of baseline characteristics for those on CKD, dialysis, and kidney transplantation *Significant p<0.05 X: SD value that cannot be calculated owing to the small number of patients (n=5) Chi-square test for categorical variables and Kruskal-Wallis test for continuous variables CKD, Chronic Kidney Disease

Variables		Total (n=39)	CKD (n=5)	On dialysis (n=12)	Underwent Kidney transplantation (n=22)	P-value
Age in Years		13.8±9.9	4.5±9	11.6±8	17.4±9.4	0.041*
Gender (Male)		18 (46.2%)	3 (75%)	6 (50%)	8 (36.4%)	0.326
Gender (Female)		21 (53.8%)	1 (25%)	6 (50%)	14 (63.6%)	
Positive Family History		11 (28.2%)	0 (0%)	3 (25%)	8 (36.4%)	0.315
Weight (kg)		39.2±15.2	46.1±X	29.5±18.4	43.5±11.9	0.046*
Height (cm)		137±17.8	161.2±X	126.5±26.4	140.7±9.0	0.108
Stunted Growth (Yes)		27 (56.4%)	1 (25%)	8 (66.7%)	18 (81.8%)	0.06
Stunted Growth (No)		8 (20.5%)	3 (75%)	3 (25%)	2 (9.1%)	
Stunted Growth (NR)		4 (10.3%)	0 (0%)	1 (8.3%)	2 (9.1%)	
Status (Deceased)		10 (25.6%)	1 (25%)	4 (33.3%)	5 (22.7%)	0.797
Status (Alive)		29 (74.4%)	3 (75%)	8 (66.7%)	17 (77.3%)	
Living City (Riyadh)		22 (56.4%)	2 (50%)	7 (58.3%)	12 (54.5%)	0.954
Living City (Outside Riyadh)		17 (43.6%)	2 (50%)	5 (41.7%)	10 (45.5%)	
Immediate-Release Cysteamine Therapy (None)		4 (10.3%)	0 (0%)	3 (25%)	0 (0%)	0.005*
Immediate-Release Cysteamine Therapy (Oral)		12 (30.8%)	1 (25%)	1 (8.3%)	10 (45.5%)	
Immediate-Release Cysteamine Therapy (Drops)		1 (2.6%)	1 (25%)	0 (0%)	0 (0%)	
Immediate-release Cysteamine Therapy (Both)		22 (56.4%)	2 (50%)	8 (66.7%)	12 (54.5%)	
Hemoglobin		103.7±22.2	93.5±26.5	107±24.2	103.3±21.3	0.578
Creatinine		253.3±245.8	300±326.5	179±211	289.5±252.9	0.436
Urea		11.6±7.3	15.4±8.2	11.1±8.7	11±6.8	0.559
Potassium		4.4±0.8	4.5±0.8	4.7±0.7	4.4±0.9	0.466
Sodium		137±3.1	135.2±3.3	136.3±2.8	138±2.8	0.114
Calcium		2.2±0.3	2.2±0.11	2.18±0.33	2.25±0.28	0.646

**Figure 1:**
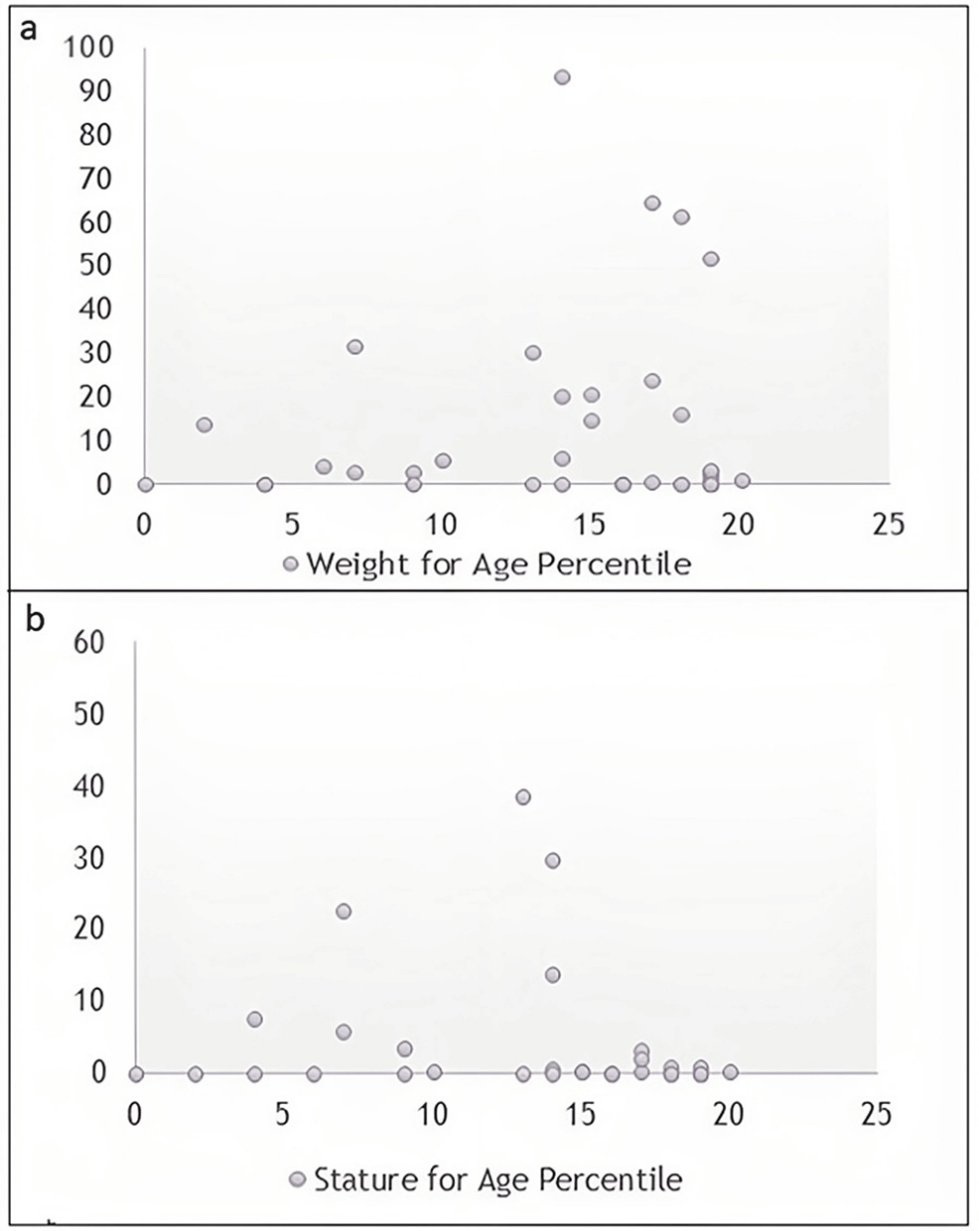
Weight and stature for age percentile a) Weight for age percentile; b) stature for age percentile

Figure 1a shows a negative correlation between weight and age, where an increase in the age percentile does not proportionately align with increases in the weight percentile, suggesting that as age increases, the relative weight percentile might not increase accordingly. On the other hand, there is no clear correlation between the age percentile and the stature percentile, as shown in Figure 1b.

Table 2 presents a comparison of height and weight for age percentile among hemodialysis and transplant patients as compared to those who were not on dialysis or had a transplant. There was no statistically significant difference between the study groups in terms of height and weight.

**Table 2:** Comparison of height and weight for age percentile according to hemodialysis and transplant status *Significant p<0.05 Mann-Whitney U test

Patient dialysis				
Parameters		Yes	No	P-value
Height for Age Percentile	Mean±SD	2.46±7.23	4.3±9.7	0.209
Weight for Age Percentile	Mean±SD	10.78±19	13.8±24.1	0.685
Transplant Patients				
Parameters		Yes	No	P-value
Height for Age Percentile	Mean±SD	1.2±2.9	7±12.7	0.746
Weight for Age Percentile	Mean±SD	14.5±25	9.4±15.8	0.286

The mortality rate in our cohort was 25.6%. Patients who died were significantly (p<0.05) younger (7±8.7 years), with a lower weight (25.9±14.1 kg), lower height (115.3±22.3 cm), and a lower hemoglobin level (91.6±26 g/L) as compared to living patients. Regarding the other demographics and laboratory values, no statistically significant difference was noted between deceased and living patients (Table 3).

**Table 3:** Comparison of baseline characteristics and laboratory values between deceased and alive patients *Significant p<0.05 Chi-square test for categorical variables and Mann-Whitney U test for continuous variables

Variables		Deceased (n=10)	Living (n=29)	P-value
Age in years		7±8.7	16.2±9.2	0.013*
Gender (Male)		7 (70%)	11 (37.9%)	0.079
Gender (Female)		3 (30%)	18 (62.1%)	
Positive Family History		1 (10%)	10 (34.5%)	0.138
Weight (kg)		25.9±14.1	42±14.2	0.007*
Height (cm)		115.3±22.3	141.6±13.3	0.027*
Stunted Growth (Yes)		6 (60%)	21 (72.4%)	0.685
Stunted Growth (No)		3 (30%)	5 (17.2%)	
Stunted Growth (NR)		1 (10%)	3 (10.3%)	
Status (Deceased)		5 (50%)	12 (41.4%)	0.635
Status (Alive)		5 (50%)	17 (58.6%)	
Living City (Riyadh)		2 (20%)	2 (6.9%)	0.457
Living City (Outside Riyadh)		4 (40%)	8 (27.6%)	
Immediate-Release Cysteamine Therapy (None)		0 (0%)	1 (3.40%)	
Immediate-Release Cysteamine Therapy (Oral)		4 (40%)	18 (62.1%)	
Immediate-Release Cysteamine Therapy (Drops)		91.6±26	107.4±19.9	0.032*
Immediate-Release Cysteamine Therapy (Both)		312.6±289	234.9±233.4	0.436
Hemoglobin		15.3±7.6	10.4±7	0.058
Creatinine		4.1±0.9	4.5±0.8	0.124
Urea		136.3±2.7	137.2±3.3	0.417
Potassium		2.15±0.36	2.26±0.24	0.309
Sodium		137±3.1	135.2±3.3	0.114
Calcium		2.2±0.3	2.2±0.11	0.646

**Figure 2:**
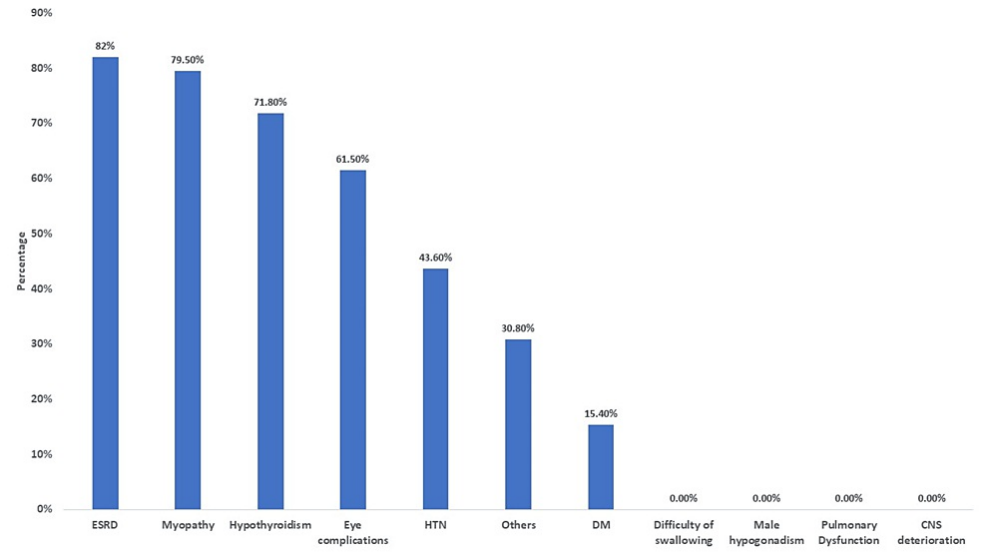
Distributions of complications ESRD, End-Stage Renal Disease; HTN, Hypertension; DM, Diabetes Mellitus; CNS, Central Nervous System

Regarding complications, the majority of patients developed myopathy (79.5%), ESRD (74.4%), and hypothyroidism (71.8%), followed by eye complications (61.5%), hypertension (43.6%), and diabetes mellitus (15.4%). However, 30.8% of patients reported other complications (Figure 2).

The univariable analysis showed that the age (OR=1.12, 95% CI: 1.02-1.22, p=0.015), weight (OR=1.1, 95% CI: 1.02-1.22, p=0.048), and height (OR=1.12, 95% CI: 1-1.21, p=0.036) were predictors of mortality in cystinosis patients. However, these risk factors did not reach statistical significance in multivariable analysis (Table 4).

**Table 4:** Univariable and multivariable logistic regression model showing the predictors of mortality in our cohort *Significant p<0.05 NE, Not Estimated

Variables	Univariable			Multivariable		
	OR	95% CI	P-value	OR	95% CI	P-value
Age, years	1.120	1.02-1.22	0.015*	1.1	0.86-1.40	0.441
Weight	1.100	1-1.21	0.048*	0.9	0.72-1.12	0.357
Height	1.120	1-1.21	0.036*	1.2	0.93-1.55	0.157
Gender	3.810	0.81-17.9	0.090	NE		
Family History	4.730	0.52-42.9	0.166	NE		
Stunted Growth	0.640	0.17-2.43	0.514	NE		
Immediate-Release Cysteamine Therapy	1.590	0.84-3.01	0.153	NE		
Patients on Dialysis	2.130	0.53-10.13	0.246	NE		
Organ Transplantation	0.550	0.13-2.4	0.430	NE		

Regarding the predictors of renal dialysis, the multivariable analysis showed that age was the independent risk factor for renal dialysis (OR=1.14, 95% CI: 1.012-1.285, p=0.031) (Table 5). Immediate-release cysteamine therapy showed a significant association in the univariable model (OR= 2.004, 95% CI: 1.003-4.008, p=0.049); however, in the multivariable, there was no significant association.

**Table 5:** Univariable and multivariable logistic regression model showing the predictors of renal dialysis in our cohort *Significant p<0.05

Variables	Univariable			Multivariable		
	OR	95% C.I.	P-value	OR	95% C.I.	P-value
Age	1.088	0.998-1.187	0.055	1.140	1.012-1.285	0.031*
Gender	1.556	0.381-6.357	0.538	0.597	0.086-4.167	0.603
Residence	1.077	0.262-4.425	0.918	1.443	0.253-8.231	0.679
Family History	0.875	0.197-3.895	0.861	0.464	0.064-3.366	0.448
Immediate-Release Cysteamine Therapy (Oral)	2.004	1.003-4.008	0.049*	1.901	0.819-4.41	0.135
Stunted Growth	2.667	0.466-15.252	0.270	2.147	0.27-17.062	0.47

Finally, when looking at the predictors of transplantation, stunted growth was found to be an independent predictor of transplantation (OR=6.624, 95% CI: 1.024-42.835, p=0.047) (Table 6).

**Table 6:** Univariate and multivariate logistic regression model showing the predictors of organ transplantation in our cohort *Significant p<0.05

Variables	Univariable			Multivariate		
	OR	95% CI	P-value	OR	95% CI	P-value
Age	0.943	0.863-1.03	0.190	0.991	0.875-1.123	0.891
Gender	0.862	0.203-3.66	0.840	0.752	0.108-5.242	0.774
Residence	1.625	0.387-6.817	0.507	1.202	0.202-7.136	0.840
Family History	0.267	0.046-1.53	0.138	0.453	0.056-3.682	0.459
Immediate-Release Cysteamine Therapy (Oral)	2.076	0.869-4.958	0.100	1.535	0.581-4.054	0.387
Stunted Growth	8.4	1.571-44.917	0.013*	6.624	1.024-42.835	0.047*

## Discussion

This study described the clinical impact of cystinosis on different organs. A substantial number of patients were found to have medical comorbidities, including CKD, being on dialysis, and undergoing kidney transplantation. Furthermore, most patients were on some form of immediate-release cysteamine therapy, either oral, eye drops, or both. Very few studies have discussed the burden of this disease and the complications patients encounter as a result of cystine accumulation.

Cystinosis was found to entail significant morbidity and mortality in our cohort, with many patients experiencing myopathy, ESRD, eye complications, and hypothyroidism. This is in line with previously published literature from the MENA region displaying similar complications in cystinosis patients [18-20]. In fact, cystinosis has been previously described as the main cause of renal Fanconi syndrome in childhood, hence entailing electrolyte disturbances, glucosuria, and hypochloremic acidosis [21]. This, in turn, results in hypercalciuric hypocalcemia that eventually leads to tetany [21]. These manifestations eventually lead to chronic renal failure and ESRD in children, as was also documented in our cohort [22,23]. In addition, growth retardation and endocrine disturbances are profound in untreated cystinosis patients that are exacerbated by ESRD [24]. Hypothyroidism and ocular manifestations of cystine deposits have also been extensively reported in cystinosis patients [21,25]. Early diagnosis of cystinosis is imperative in preventing the consequences of cystine accumulation and allowing early treatment. Due to the rarity of this disease, it is generally overlooked when patients present with vague multiorgan symptoms. In addition, one could postulate that, due to its rarity, there exist several differential diagnoses that allow it to be missed by clinicians [26]. In one case report, a 15-month-old boy presented with biochemical findings of Fanconi syndrome and was prescribed an electrolyte supplement with no further workup. However, at the age of six, the patient's condition worsened as he developed photophobia and renal failure with a delayed diagnosis of cystinosis [9]. The patient later passed away due to renal failure. Furthermore, delayed diagnosis and management of nephropathic cystinosis with ocular involvement have been shown to cause complete loss of vision if not treated promptly [7].

Cystinosis can cause significant morbidities, which may have an adverse impact on the quality of life. However, treatment options aimed at relieving these symptoms do exist. As a first line, clinicians aim at treating fluid and electrolyte losses due to Fanconi syndrome with fluid and electrolyte replacements [21]. ESRD disease is addressed by hemodialysis while patients wait for renal transplants [21]. However, a breakthrough in cystinosis treatment was the discovery of cystine depletion by cysteamine [27]. Immediate-release cysteamine therapy was found to retard the rate of kidney damage, improve linear growth, and decrease cystine damage to different organs [28-31]. These findings resulted in immediate-release cysteamine therapy becoming the treatment of choice for pre-transplant cystinosis after receiving Food and Drug Administration (FDA) approval in 1994 [21]. In contrast, other studies have shown that topical immediate-release cysteamine eye drops have limited effects in decreasing corneal cystine deposits [32]. Nevertheless, 90% of our patients were on some form of immediate-release cysteamine therapy.

Stratifying the patients based on the predictors of complications is invaluable in clinicians' decision-making process [3]. In our analysis, age was found to be a predictor of renal dialysis in cystinosis patients. In addition, stunted growth was found to be an independent predictor of transplantation. These predictors of complications are of limited utility due to the small sample size. Further studies with larger sample sizes are needed to identify the predictors of complications in cystinosis patients.

Previous studies assessing the overall burden of cystinosis were conducted outside Saudi Arabia [3]. Few studies looking at genetic typing of cystinosis and eye manifestations of cystinosis have been addressed in Saudi Arabia; however, a large sample comprehensive analysis of overall complications and prediction models has not been previously attempted [7,8]. Cystinosis is caused by mutations in the CTNS gene that codes for the protein cystinosin [33]. CTNS gene mutations display genetic polymorphism and mutational variability between different ethnic groups, hence phenotypically displaying varying disease severity [8]. European and Middle Eastern cystinosis patients have been found to carry a 57-kB genomic deletion, causing CTNS loss of function [34,35]. However, studies in Egyptian, Turkish, Tunisian, and Jordanian cystinosis patients demonstrated the absence of this 57-kB genomic deletion and the prevalence of other novel mutations [23,36-38]. In Saudi Arabia, Aldamesh et al. identified eight mutations in 21 children with cystinosis, four of which were novel (c.530A>G, c.681G>A, 1013T>G, and c.1018_1041del) [8]. This study concluded that these mutations should provide the basis for routine molecular diagnosis of cystinosis in Saudi Arabia [8]. A large percentage of patients with cystinosis in Saudi Arabia are followed up at the King Faisal Specialist Hospital in Riyadh. This enabled us to capture the majority of cystinosis patients in the kingdom with 39 reported cases, which is a relatively large sample at a single center when considering the rarity of this disease.

We acknowledge that this study has some limitations. First, the retrospective nature of this study fails to account for several confounders that were not reported, such as family history and leukocyte cystine levels. Second, the small sample size limits the generalizability of this study. Furthermore, long-term follow-ups are required to fully assess the burden of cystinosis and the prognosis of these patients.

Future directions

Future research on cystinosis, particularly in Saudi Arabia and other Arab countries, should focus on expanding epidemiological studies to better understand the prevalence and genetic diversity of the disease. Longitudinal studies are needed to assess long-term patient outcomes and the efficacy of current treatments such as immediate-release cysteamine therapy. Investigating new therapeutic approaches, including personalized medicine and gene therapy, could offer improved patient care. Additionally, studies should explore the quality of life, psychosocial impacts, and the effectiveness of educational and awareness campaigns. Comparative studies between different populations and the integration of advanced diagnostic and monitoring technologies could provide deeper insights into the disease. Ultimately, these efforts should inform healthcare policies and interventions to enhance patient support and treatment access.

## Conclusions

In conclusion, the high prevalence of CKD, dialysis, and kidney transplantation emphasizes the significant renal impact of the disease. Most patients were treated with a combination of oral and eye drop cysteamine therapy. The mortality rate was notably high, with deceased patients being younger and having lower weight, height, and hemoglobin levels. Complications such as myopathy, ESRD, and hypothyroidism were common. Age was identified as an independent risk factor for renal replacement therapy, and stunted growth emerged as a significant predictor of transplantation. These findings highlight the critical need for early diagnosis, vigilant monitoring, and a multidisciplinary approach to management in cystinosis patients to improve outcomes and reduce mortality. Further studies with larger cohorts are required to clearly illustrate the burden of this disease and to determine independent predictors of cystinosis to help guide management and determine prognosis.
